# Urine Osmolarity and Risk of Dialysis Initiation in a Chronic Kidney Disease Cohort – a Possible Titration Target?

**DOI:** 10.1371/journal.pone.0093226

**Published:** 2014-03-27

**Authors:** Max Plischke, Maria Kohl, Lise Bankir, Sascha Shayganfar, Ammon Handisurya, Georg Heinze, Martin Haas

**Affiliations:** 1 Division of Nephrology and Dialysis, Department of Internal Medicine III, Medical University Vienna, Vienna, Austria; 2 Section for Clinical Biometrics, Center of Medical Statistics, Informatics and Intelligent Systems, Medical University of Vienna, Vienna, Austria; 3 INSERM UMRS 1138, Equipe 2, Centre de Recherche des Cordeliers, Paris, France; 4 Division of Cardiology, Pulmonology, and Vascular Medicine, Medical Faculty, University Hospital Düsseldorf, Düsseldorf, Germany; Medical University of Graz, Austria

## Abstract

**Background:**

Increasing evidence is linking fluid intake, vasopressin suppression and osmotic control with chronic kidney disease progression. Interestingly, the association between urine volume, urine osmolarity and risk of dialysis initiation has not been studied in chronic kidney disease patients before.

**Objective:**

To study the relationship between urine volume, urine osmolarity and the risk of initiating dialysis in chronic kidney disease.

**Design:**

In a retrospective cohort analysis of 273 patients with chronic kidney disease stage 1–4 we assessed the association between urine volume, urine osmolarity and the risk of dialysis by a multivariate proportional sub-distribution hazards model for competing risk data according to Fine and Gray. Co-variables were selected via the purposeful selection algorithm.

**Results:**

Dialysis was reached in 105 patients over a median follow-up period of 92 months. After adjustment for age, baseline creatinine clearance, other risk factors and diuretics, a higher risk for initiation of dialysis was found in patients with higher urine osmolarity. The adjusted sub-distribution hazard ratio for initiation of dialysis was 2.04 (95% confidence interval, 1.06 to 3.92) for each doubling of urine osmolarity. After 72 months, the estimated adjusted cumulative incidence probabilities of dialysis were 15%, 24%, and 34% in patients with a baseline urine osmolarity of 315, 510, and 775 mosm/L, respectively.

**Conclusions:**

We conclude that higher urine osmolarity is associated with a higher risk of initiating dialysis. As urine osmolarity is a potentially modifiable risk factor, it thus deserves further, prospective research as a potential target in chronic kidney disease progression.

## Introduction

Medicinal use of water in chronic kidney disease (CKD) has gained research interest lately [1], as established efforts to retard CKD progression remain far from satisfactory [2]. Epidemiological data associating fluid intake or urine volume with GFR decline in humans have not been fully conclusive [3]–[7]. Nonetheless, there is increasing evidence linking fluid intake, vasopressin suppression and osmotic control with CKD and ADPKD progression [8]–[12]. Kidney excretion is adjusted according to water and dietary solute intake, as well as water and solute losses by lungs, skin, and the gastrointestinal tract. The required urine volume can be determined by dividing the daily osmolar excretion, to maintain the body's solute content at steady state, by the maximal urine osmolality, with failing kidneys losing capacity to concentrate urine maximally. As such, water intake required to achieve comparable urinary solute dilution varies considerably between individuals. [1]

Interestingly, median 24-hour urine osmolality is greater than that of plasma in humans, suggesting continuous antidiuretic action [13], which has been associated with renal function decline [10]. Consequentially, Wang et al. recently devised a quantitative method to determine the amount of water needed on a case-by-case basis to achieve a mean urine osmolality equivalent to that of plasma [13]. Relationships between urine osmolarity (given as mosm/L compared to mosm/kg H_2_O for osmolality) and GFR decline have been described in two studies [3], [7] with contrasting results. We were interested in studying urine volume and urine osmolarity in terms of harder endpoints in chronic kidney disease. Thus we set out to study these variables in terms of risk of initiating dialysis, with death as a competing event.

## Subjects and Methods

### Patients

All patients attending our nephrology outpatient department between 1 January 2000 and 31 December 2002 were included in a single-centre cohort study. The study baseline was defined as one year after the first visit, while the time period between the first visit and baseline was defined as the run-in phase. Baseline demographic data for each patient were collected from outpatient files including medication, co-morbidities, and the nature of renal disease. A minimum of two visits, with 24-hour urine samples taken before and after baseline, were defined as inclusion criteria. Exclusion criteria were a reported urine volume less than 500 ml/d or a creatinine clearance below 15 ml/min (CKD 5). The mean of all measurements taken during the run-in phase (median: 5 [25^th^–75^th^ percentiles: 3–8]) was used as the baseline value for each parameter.

The primary endpoint of the study was time to dialysis, with death as the competing event. Mortality data and data on the initiation of dialysis until 31 December 2008 were obtained from Statistics Austria (the national statistics institution) and the Austrian Dialysis and Transplantation Registry (ÖDTR), respectively. Patients starting dialysis had no loss of follow-up according to ÖDTR. Because of a possible relocation of a patient to a country other than Austria, a minor loss of follow-up for mortality data from Statistics Austria cannot be excluded.

### Ethics Statement

This was a retrospective study making use of data already collected during routine patient care at our outpatient department. The processing and analysis of data was done after anonymization. Therefore no informed consent was requested from patients. This approach was reviewed and approved by the local ethics committee (Ethikkomission Medizinische Universität Wien).

### Laboratory data

Standard 24-hour urine samples of the patients were analysed in regard of proteinuria, creatinine, sodium, urea nitrogen, and potassium levels, in accordance with routinely used methods at our central laboratory (Clinical Institute of Medical and Chemical Laboratory Diagnostics, Medical University of Vienna). On the day of each visit, serum samples were analysed for creatinine, sodium, potassium, glucose and urea nitrogen levels in accordance with routine methods.

After conversion of glucose and urea nitrogen from mg/dl in mmol/L the estimated urine osmolarity (U_osm_) (mosm/L) was calculated as follows: 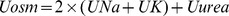
Estimated plasma osmolarity (P_osm_) (mosm/L) was calculated as follows: 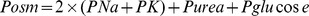
U_Na_, U_K_, and U_urea_ are the concentrations of sodium, potassium and urea in the urine (all in mmol/L), and P_Na_, P_K_, P_urea_, and P_glucose_ are the concentrations of sodium, potassium, urea and glucose in plasma (in mmol/L).

### Statistical analysis

Continuous variables were described by medians (25^th^ to 75^th^ percentiles), and compared between groups using Wilcoxon's rank sum tests. Correlations between continuous variables were assessed by Spearman's rank correlation coefficient. For further analysis, osmolarity, proteinuria and creatinine clearance were log-base-2 transformed because of the skewed distributions of these variables. To describe intra- versus inter-individual variance of urine osmolarity, we conducted a variance component analysis including all run-in urine osmolarity values that were available for each patient using a mixed model with patients as levels of a random factor. The outcome variable was time to dialysis, with death as the competing event. Patients who were alive without dialysis at the time of their last visit were censored. Absolute event rates were computed as the number of events divided by the total follow-up time for all patients. Observations with missing values were not used in the calculated models. We described the distribution of time to dialysis using cumulative incidence functions, and compared groups using Gray's test [14].

Due to the established relationship between baseline creatinine clearance and risk of initiating dialysis/ESRD, and the known progressive loss in urine concentration ability with decreasing renal function [1], it seemed important to introduce creatinine clearance as an adjustment factor in all further analyses.

We fitted two multivariate proportional sub-distribution hazards models for competing risk data according to Fine and Gray [15] in order to assess the effect of urine osmolarity or volume on the risk for initiating dialysis. In these models, we considered osmolarity or urine volume and included those variables that either proved significant in a multivariate model (P<0.10) or changed the log hazard ratio of osmolarity or urine volume by more than 15% when those variables were excluded from the analyses (purposeful selection algorithm) [16]. We assumed that any variable not selected would have no relevant impact on our conclusions. All variables listed in Table 1 (except 24-hour proteinuria, 24-hour osmolar excretion and 24-hour sodium excretion) were considered as potential confounders. Results from multivariate competing risk regression were described by means of sub-distribution hazard ratios (SHR) and 95% confidence intervals (95% CI), and by computing and visualising estimated cumulative incidence curves at specific covariate values. As urine osmolarity and creatinine clearance were log-base-2 transformed, their SHR correspond to each doubling of these variables. We checked for significant pairwise interactions of variables and for time-dependent effects by including interactions with follow-up time. Non-linear effects were assessed by the method of fractional polynomials [17]. For sensitivity analysis, we also estimated a cause-specific (death-censored) Cox regression model. The R-software, version 2.12 (www.r-project.org), was used for statistical analysis.

**Table 1:** Demographic and clinical characteristics of all patients at baseline, and in the subgroups of CKD patients stages 1-3a (creatinine clearance ≥45 ml/min) and stage 3b-4 (creatinine clearance ≥15 and <45 ml/min).

	Total	CKD 1-3a	CKD 3b-4
	(n = 273)	(n = 141)	(n = 132)
Age (years)	56 (42–67)	50 (37–61)	59 (46–71)
Male	153 (56%)	87 (62%)	66 (50%)
BMI	26 (23–30)	26 (23–30)	26 (23–30)
CCl (ml/min)	48 (30–79)	78 (60–105)	30 (25–35)
MAP (mmHg)	97 (93–102)	97 (93–103)	98 (94–102)
P_osm_ (mosm/L)	308 (302–315)	303 (300–308)	313 (308–318)
			
Urine analysis			
Proteinuria (g/L)	0.87 (0.24–2.34)	0.76(0.21–2.34)	1.01 (0.26–2.31)
Proteinuria 24 h (g/24 h)	2.05 (0.53–5.63)	1.72 (0.53–5.2)	2.21 (0.57–6.12)
Volume (ml/24 h)	2220 (1935–2871)	2157 (1839–2700)	2325 (2000–3000)
U_osm_ (mosm/L)	510 (414–622)	607 (477–740)	445 (370–519)
Osmolar excretion (mosm/24 h)	1200 (930–1412)	1309 (1091–1636)	1018 (810–1254)
Sodium (mmol/L)	84 (68–104)	96 (75–116)	75 (61–93)
Sodium excretion (mmol/24 h)	201 (141–251)	215 (167–265)	181 (134–234)
			
Underlying kidney disease			
Polycystic kidney disease	18 (7%)	6 (4%)	12 (9%)
Diabetic nephropathy	20 (7%)	7 (5%)	13 (10%)
Glomerular disease	98 (36%)	73 (52%)	25 (19%)
Other	11 (4%)	4 (3%)	7 (5%)
Unknown	126 (46%)	51 (36%)	75 (57%)
			
Comorbidities			
Chronic heart failure	8 (3%)	3 (2%)	5 (4%)
Diabetes mellitus	59 (23%)	23 (18%)	36 (27%)
Liver cirrhosis (child C)	0 (0%)	0 (0%)	0 (0%)
			
Medication			
ACEI/AT-II Blocker	216 (85%)	111 (85%)	105 (84%)
Diuretics	120 (47%)	45 (35%)	75 (60%)
Beta-blocker	119 (47%)	47 (36%)	72 (58%)
Non-dihydropyridine CCB	39 (15%)	21 (16%)	18 (14%)

Abbreviations: BMI, body mass index; CCl, creatinine clearance; MAP, mean arterial pressure; P_osm_, plasma osmolarity; U_osm_, urine osmolarity; ACEI/AT-II, angiotensin converting enzyme/angiotensin II receptor antagonist; CCB, calcium channel blocker. Values are given as median (Q1-Q3), if not stated otherwise.

## Results

### Baseline data

Three hundred and seventy-two patients were examined for eligibility. After applying all inclusion and exclusion criteria, a total of 273 patients (56% male) with CKD class 1-4 and a median age of 56 years (42 to 67 years) were confirmed eligible and included in the study (**Table 1**). Median creatinine clearance was 48 (30 to 79) ml/min. Kidney disease was unknown in 46% of the patients; the remaining patients had mainly polycystic kidney disease, different forms of glomerulonephritis, or diabetic nephropathy (Table 1). Nearly all patients received antihypertensive drugs with an effect on protein excretion, such as inhibitors of the renin angiotensin aldosterone system, non-dihydropyridine calcium channel blockers, or beta-blockers.

There was a significant inverse correlation between average run-in 24-hour urine volume and average run-in urine osmolarity (R = -0.45; P<0.001). Urine osmolarity was significantly higher in men than in women (522 [446 to 629] vs. 458 [385 to 596] mosm/L; P<0.01), in patients without diuretics (520 [422 to 679] vs. 490 [408 to 577] mosm/L; P<0.05), and in patients without beta-blocker therapy (526 [429 to 673] vs. 463 [392 to 557] mosm/L; P<0.01). Urine osmolarity and creatinine clearance were positively correlated (R = 0.6, p<0.01). The total variance for run-in urine osmolarity was 45253; the intra-individual variance was about one fourth (σ2 = 11349), i.e., random fluctuation within a patient explained 25% of the total variance, while the inter-individual variance component explained about 75% of the total variance. One patient was missing data for proteinuria, and 20 patients each were missing data for diuretics and beta-blocker therapy.

### Follow–up

Median follow-up until death or censoring was 92 (76 to 95) months. End-stage renal disease developed in 105 patients (39%). Thirty-eight patients (14%) died on dialysis and 35 patients (13%) died with functioning kidneys.

The absolute event rate for ESRD was 0.07/year. Event rates were 0.05/year for patients with mild to moderate chronic kidney disease (CKD stage 1–3, creatinine clearance ≥30 ml/min), and 0.22/year for those with severe CKD (stage 4, creatinine clearance 15–29 ml/min).

### Urine osmolarity and risk of initiating dialysis

Univariate analysis, without adjustment for baseline creatinine clearance (see correlation above), suggested a higher cumulative incidence of dialysis in patients with lower-than-median urine osmolarities (p<0.01). Multivariate competing risk regression analysis, with death as the competing risk, adjusted for age, creatinine clearance, proteinuria, type of underlying renal disease, beta-blocker and diuretic therapies, showed that a higher urine osmolarity was associated with a higher risk of initiating dialysis (**Table 2**).

**Table 2:** The independent effect of urine osmolarity, age, protein excretion, kidney function, renal disease and different drugs, on the risk of initiating dialysis in the competing risk regression analysis.

	SH Ratio	95% confidence interval		p-value
Urine osmolarity (per doubling)	2.04	1.06	3.92	0.03
Age (per decade)	0.87	0.74	1.02	0.08
Proteinuria (per doubling)	1.85	1.60	2.13	<0.001
Creatinine clearance (per doubling)	0.15	0.09	0.23	<0.001
Renal disease (PKD vs. other renal diseases)	3.44	1.73	6.81	<0.001
Beta-blocker therapy (yes vs. no)	1.54	0.97	2.43	0.07
Diuretic therapy (yes vs. no)	1.62	1.03	2.55	0.04

Abbreviations: SH Ratio, subdistribution hazard ratio; PKD, polycystic kidney disease

**Figure 1:**
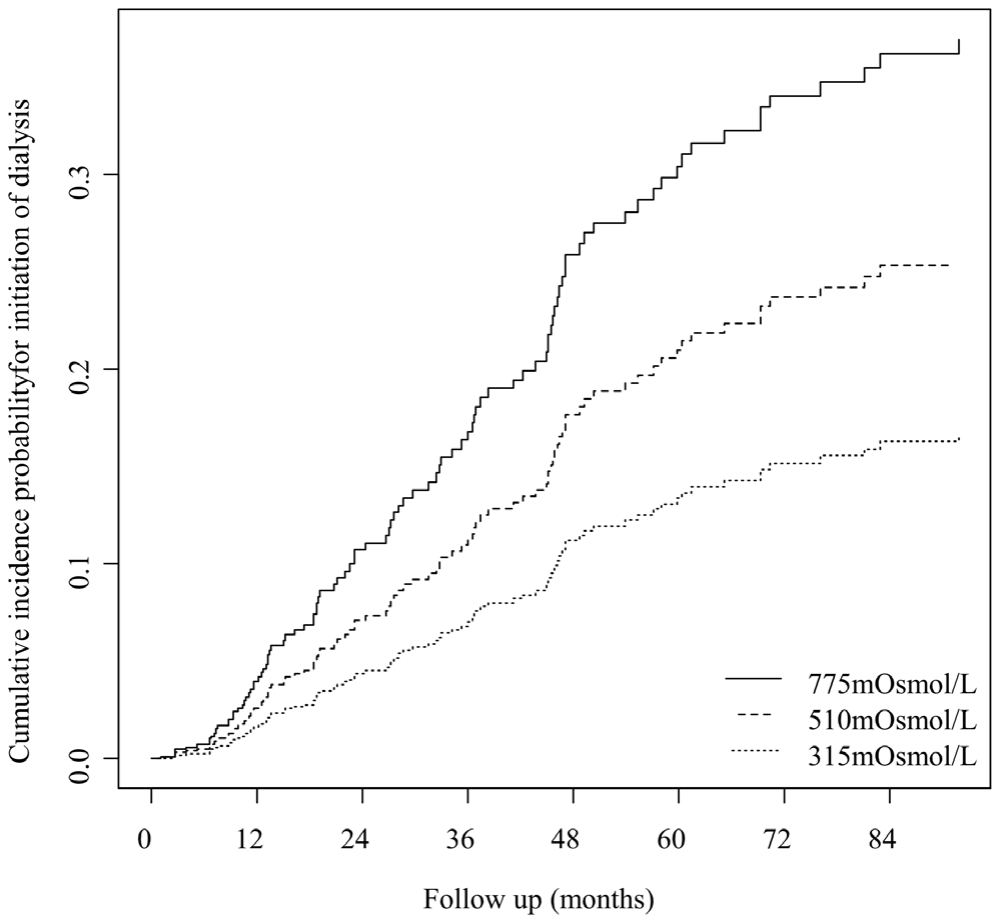
Cumulative incidence probabilities of dialysis initiation for different baseline urine osmolarities. Cumulative incidence probabilities of dialysis initiation for a baseline urine osmolarity of 315, 510 or 775/L (10^th^, 50^th^, and 90^th^ percentile), estimated from the proportional sub-distribution hazards model. Given the estimated adjusted subdistribution hazard ratio (SHR) of 2.04 (p = 0.033) per doubling of urine osmolarity, the SHRs comparing patients with 775 mosm/L or 315 mosm/L to patients with 510 mosm/L were 1.54 (95%CI:1.03 to 2.28) or 0.61 (95%CI: 0.39 to 0.96), respectively.

Based on this model, we estimated the adjusted cumulative incidence probabilities of dialysis for patients with three different urine osmolalities (10^th^, 50^th^ and 90^th^ percentile), assuming average values for all other covariates. A constant and stepwise significant increase was seen in patients with low (315 mosm/L), intermediate (510 mosm/L), and high (775 mosm/L) baseline urine osmolarity (**Figure 1**; p<0.05). At 72 months, the estimated cumulative incidence probabilities of dialysis in these patients were 15%, 24% and 34%, respectively. Lower baseline creatinine clearance, higher baseline protein excretion, the type of underlying renal disease, and treatment with diuretics were also independently associated with a higher risk of dialysis (Table 2). No significant interactions of urine osmolarity with other variables in the model were found. There was no evidence of time-dependent effects or a non-linear effect of urine osmolarity or any other metric covariate. The cause-specific Cox model yielded a similar result for the adjusted effect of urine osmolarity (cause-specific hazard ratio 2.19, 95% CI 1.21 to 3.95).

### Urine volume and risk of initiating dialysis

There was a significant inverse association between average 24-hour urine volume and average urine osmolarity (R = −0.46; P<0.01). Therefore, we did not adjust for urine osmolarity in the multivariate regression analysis for urine volume. In this model, higher protein excretion, lower creatinine clearance, and the underlying renal disease but not urine volume were associated with a higher risk of dialysis (**Table 3**).

**Table 3:** The independent effect of urine volume, age, protein excretion, kidney function, renal disease and different drugs, on the risk of of initiating dialysis in the competing risk regression analysis.

	SH Ratio	95% confidence interval		p-value
Urine volume (per 0.5 L/d)	1.05	0.92	1.20	0.49
Age (per decade)	0.90	0.77	1.05	0.18
Proteinuria (per doubling)	1.79	1.54	2.07	<0.001
Creatinine clearance (per doubling)	0.20	0.13	0.30	<0.001
Renal disease (PKD vs. other renal diseases)	3.76	1.92	7.34	<0.001
Beta-blocker therapy (yes vs. no)	1.58	0.98	2.54	0.06
Diuretic therapy (yes vs. no)	1.59	0.99	2.54	0.05

Abbreviations: SH Ratio, subdistribution hazard ratio; PKD, polycystic kidney disease

## Discussion

The present study demonstrates an independent, positive relationship between urine osmolarity and risk of initiating dialysis in a cohort of CKD patients stage 1 through 4. Competing risk models were adjusted for age, creatinine clearance, proteinuria, type of underlying renal disease, beta-blocker and diuretic therapies.

Two published studies have described relationships between 24-h urine osmolarity and GFR change over time. Hebert et al. reported a significant inverse relationship between baseline urine osmolarity and GFR decline in non-polycystic kidney disease patients (subgroup of full cohort), adjusted for diet, blood pressure and body surface area. Adjusting for additional covariates such as baseline GFR or diuretics use failed to result in statistically significant models. Other models were calculated for follow-up urine osmolarities (collected after the study baseline; thus, not fully comparable) and did stay significant after further adjustments. [3] Torres et al. in turn suggested that higher baseline 24-h Uosm was associated with higher GFR decline across time in a cohort of ADPKD patients. [7]

The association between urine osmolarity and the risk of initiating dialysis, a stronger marker for end stage renal disease, has not been previously investigated. Our data thus adds to the existing evidence. The data is in line with Torres et al. suggesting a faster renal function decline in patients with higher urine osmolarity. Our cohort appeared to have the highest urine osmolarity with a median of 510 (IQR: 414–622) mosm/L versus a mean of 368±159 mosm/L (Torres et al.), and a mean of 270 to 334 mosm/L (depending on protein diet group and polycystic kidney disease status) (Hebert et al.). It is therefore conceivable that lowering urine osmolarity below a specific threshold might be harmful to the kidney as well. A recent study addressing the association between sodium excretion, an important part of total urine osmolarity, and end-stage renal disease in patients with type 1 diabetes mellitus showed an inverse association with end stage renal disease [18]. Furthermore, a U-shaped curve was described for sodium excretion and mortality, such that subjects with the highest and the lowest sodium excretion had the highest mortality, partly supporting the hypothesis of low urine osmolarity being associated with harmful effects. Interestingly, in the univariate versus the multivariable analysis the strong effect of impaired renal function seems to obscure the positive correlation of osmolarity with the incidence of ESRD. Adjusting for creatinine clearance reveals this relationship. We conclude that among patients with equal renal function those with higher osmolarity values are more likely to progress to ESRD than those with lower osmolarity values in our cohort.

As described in the methods, estimated urine osmolarity was calculated from urine sodium, potassium and urea concentrations in 24 hour urine samples. These are the dominant solutes in the urine (plus the anions associated with sodium and potassium). Other solutes represent less than 10% of total urinary solutes (in the absence of glucosuria). Thus, estimated urine osmolarity is often used as an approximation of true osmolarity. Regarding the difference between osmolality (in mosm/kg H_2_O) and osmolarity (in mosm/L), it is negligible in our study because the density of urine is very close to that of pure water in the range of values considered here. Other authors have used estimated urine osmolarity in several studies. [3], [19]

Several authors have studied urine volume and GFR decline. Opposed to the prevailing view that water is beneficial in CKD, Hebert et al. reported higher GFR decline in higher urine volume quartiles. However, multivariable adjustment seemed to diminish the association.[1], [3] In support of Hebert et al., Wang et al. found a weak, but significant association between higher urine volume and GFR decline.[4] A large study by Clark et al. showed a relationship between higher urine volume and lower rate of eGFR decline, which stayed significant after multivariable adjustment. [5] Another large study reported worse kidney function in individuals with a lower self-reported fluid intake [6]; however, neither urine volume nor urine osmolarity were evaluated. It has been suggested that lower mean baseline GFR in cohorts of Hebert et al. and Wang et al., which is associated with alterations in water metabolism, might explain these paradoxical findings. With falling GFR, the ability to concentrate urine to osmolalities greater than that of plasma is progressively lost. [1] As such, in contrast to the general population, solute excretion and urine volume are closely interrelated in severe chronic kidney disease [20], [21]. In our study we did not find a significant relationship between urine volume and a higher risk of dialysis. The rather weak association between urine volume and 24-hour urine osmolarity in the present study suggests that, in contrast to patients with severe kidney failure, urine concentration by vasopressin was still effective in the vast majority of the patients.

Stimulation of vasopressin secretion is supposed to be the cause of the more rapid decline of kidney function in patients with a high urine osmolality. Vasopressin exerts a range of different effects and interacts through the three receptors V1a, V2 and V1b [22]. The antidiuretic effect is mainly mediated by the V2 receptor and includes increased tubular permeability for water and urea, and stimulation of ENaC-mediated sodium reabsorption [22]. Chronic administration of vasopressin in rats was shown to increase renal blood flow, glomerular filtration rate, and renal mass [23]–[25]. Vice versa, prevention of hyperfiltration in 5/6 nephrectomised rats by chronic inhibition of vasopressin secretion led to less glomerular sclerosis, less interstitial fibrosis and slower progression of renal failure [8], [9], [26]. However, precise plasma levels of vasopressin are difficult to obtain. Furthermore, non-detectable changes of vasopressin lead to a broad range of different urine osmolalities [22].

The optimal range of urine osmolality is difficult to define. Studies in normal rats and healthy humans have shown that urine concentration above an osmolarity of about 300 mosm/L induces a significant hyperfiltration [22], [27]. In accordance with these findings we registered the lowest risk of initiation dialysis in patients with a urine osmolarity of a similar range. On the other hand we cannot rule out that urine osmolarity values below those in our cohort range might worsen the risk of renal function decline as suggested by the cohort of Hebert et al. (see above).

Either increasing fluid intake or decreasing the intake of osmolytes could achieve a reduction of urine osmolality. A recently published formula could be used to estimate the quantity of fluid needed to achieve a urine osmolality equivalent to that of plasma [13]. An alternative approach might be the use of vaptans, which suppress vasopressin activity by antagonistic binding to the VP receptors. Recently a protective effect of dual V1a/V2 blockade on the progression of CKD has been reported in rats [28]. A similar effect has been shown in diabetic rats, where the rise in albuminuria was prevented by a V2 antagonist [29].

Our study is limited by its design. As an observational cohort study can only prove associations and not causality, it remains to be proven in a prospective trial that changing urine osmolarity indeed has a positive effect on rate of renal function decline in CKD, before any therapeutic recommendation can be made. Furthermore, our study cohort showed a high event rate for ESRD, which might be explained by the cohort's relatively high baseline proteinuria. Thus, it is not clear if the study results are applicable to CKD populations with different demographics. Nonetheless, our study further strengthens the link between urine osmolarity and renal function decline by establishing a relationship with risk of dialysis initiation. In addition to the hard end-point of ESRD, a long follow-up period, the use of baseline variables issued from several measurements over a 1 year run-in phase reducing the impact of possible occasional sampling errors, and the application of competing risk analysis techniques strengthen the conclusions of this study.

While it is indisputable that, in the presence of a competing risk such as death, cumulative incidence curves are the method of choice rather than conventional Kaplan-Meier estimates, there is some controversy as to whether the standard cause-specific (death-censored) Cox regression analysis or the proportional sub-distribution hazards (Fine-Gray) model should be used to obtain adjusted hazard ratios. We decided to use the latter because it directly models differences in cumulative incidences and permits the investigator to predict the cumulative incidence of ESRD based on the covariate values of a patient. This would not have been possible when using the cause-specific Cox model. Our competing risk analysis using the Fine-Gray model mirrors more precisely the association between a covariate and the cumulative incidence of ESRD in patients at high risk of death during the observation period. Our observation that urine osmolarity might be positively associated with the risk for ESRD is robust as regards the type of analysis used, as results of the cause-specific Cox model were very similar.

In conclusion, we demonstrate that higher urine osmolarity is independently associated with a higher risk of initiating dialysis in a cohort of patients with CKD stage 1 to 4. Modifying urine osmolarity by dietary counselling or pharmaceutical interventions might evolve into a further treatment option in ESRD.

## References

[1] Wang CJ, Grantham JJ, Wetmore JB (2013) The medicinal use of water in renal disease. Kidney Int 84: 45–53. 23423255 10.1038/ki.2013.23

[2] Bankir L, Bouby N, Ritz E (2013) Vasopressin: a novel target for the prevention and retardation of kidney disease? Nat Rev Nephrol 9: 223–239. 23438973 10.1038/nrneph.2013.22

[3] Hebert LA, Greene T, Levey A, Falkenhain ME, Klahr S (2003) High urine volume and low urine osmolality are risk factors for faster progression of renal disease. Am J Kidney Dis 41: 962–971. 12722030 10.1016/s0272-6386(03)00193-8

[4] Wang X, Lewis J, Appel L, Cheek D, Contreras G, et al (2006) Validation of creatinine-based estimates of GFR when evaluating risk factors in longitudinal studies of kidney disease. J Am Soc Nephrol 17: 2900–2909. 16988064 10.1681/ASN.2005101106

[5] Clark WF, Sontrop JM, Macnab JJ, Suri RS, Moist L, et al (2011) Urine volume and change in estimated GFR in a community-based cohort study. Clin J Am Soc Nephrol 6: 2634–2641. 21885793 10.2215/CJN.01990211 PMC3359569

[6] Strippoli GF, Craig JC, Rochtchina E, Flood VM, Wang JJ, et al (2011) Fluid and nutrient intake and risk of chronic kidney disease. Nephrology (Carlton) 16: 326–334. 21342326 10.1111/j.1440-1797.2010.01415.x

[7] Torres VE, Grantham JJ, Chapman AB, Mrug M, Bae KT, et al (2011) Potentially modifiable factors affecting the progression of autosomal dominant polycystic kidney disease. Clin J Am Soc Nephrol 6: 640–647. 21088290 10.2215/CJN.03250410 PMC3082424

[8] Bouby N, Bachmann S, Bichet D, Bankir L (1990) Effect of water intake on the progression of chronic renal failure in the 5/6 nephrectomized rat. Am J Physiol 258: F973–979. 2184677 10.1152/ajprenal.1990.258.4.F973

[9] Sugiura T, Yamauchi A, Kitamura H, Matsuoka Y, Horio M, et al (1999) High water intake ameliorates tubulointerstitial injury in rats with subtotal nephrectomy: possible role of TGF-beta. Kidney Int 55: 1800–1810. 10231442 10.1046/j.1523-1755.1999.00443.x

[10] Meijer E, Bakker SJ, de Jong PE, Homan van der Heide JJ, van Son WJ, et al (2009) Copeptin, a surrogate marker of vasopressin, is associated with accelerated renal function decline in renal transplant recipients. Transplantation 88: 561–567. 19696640 10.1097/TP.0b013e3181b11ae4

[11] Meijer E, Bakker SJ, Halbesma N, de Jong PE, Struck J, et al (2010) Copeptin, a surrogate marker of vasopressin, is associated with microalbuminuria in a large population cohort. Kidney Int 77: 29–36. 19847155 10.1038/ki.2009.397

[12] Nagao S, Nishii K, Katsuyama M, Kurahashi H, Marunouchi T, et al (2006) Increased water intake decreases progression of polycystic kidney disease in the PCK rat. J Am Soc Nephrol 17: 2220–2227. 16807403 10.1681/ASN.2006030251

[13] Wang CJ, Creed C, Winklhofer FT, Grantham JJ (2011) Water prescription in autosomal dominant polycystic kidney disease: a pilot study. Clin J Am Soc Nephrol 6: 192–197. 20876670 10.2215/CJN.03950510 PMC3022242

[14] Gray RJ (1988) A class of K-sample tests for comparing the cumulative incidence of a competing risk. The annals of statistics: 1141–1154.

[15] Fine JP, Gray RJ (1999) A proportional hazards model for the subdistribution of a competing risk. Journal of the American statistical association: 496–509.

[16] Hosmer DW, Lemeshow S, May S (2008) Applied Survival Analysis: Regression Modeling of Time to Event Data. Chickester, UK: Wiley.

[17] Royston P, Altman DG (1994) Regression using fractional polynomials of continuous covariates: parsimonious parametric modelling. Applied Statistics: 429–467.

[18] Thomas MC, Moran J, Forsblom C, Harjutsalo V, Thorn L, et al (2011) The association between dietary sodium intake, ESRD, and all-cause mortality in patients with type 1 diabetes. Diabetes Care 34: 861–866. 21307382 10.2337/dc10-1722 PMC3064042

[19] Perucca J, Bouby N, Valeix P, Bankir L (2007) Sex difference in urine concentration across differing ages, sodium intake, and level of kidney disease. Am J Physiol Regul Integr Comp Physiol 292: R700–705. 16990487 10.1152/ajpregu.00500.2006

[20] Feinfeld DA, Danovitch GM (1987) Factors affecting urine volume in chronic renal failure. Am J Kidney Dis 10: 231–235. 3631070 10.1016/s0272-6386(87)80179-8

[21] Luft FC, Fineberg NS, Sloan RS, Hunt JN (1983) The effect of dietary sodium and protein on urine volume and water intake. J Lab Clin Med 101: 605–610. 6833831

[22] Bankir L (2001) Antidiuretic action of vasopressin: quantitative aspects and interaction between V1a and V2 receptor-mediated effects. Cardiovasc Res 51: 372–390. 11476728 10.1016/s0008-6363(01)00328-5

[23] Bouby N, Ahloulay M, Nsegbe E, Dechaux M, Schmitt F, et al (1996) Vasopressin increases glomerular filtration rate in conscious rats through its antidiuretic action. J Am Soc Nephrol 7: 842–851. 8793792 10.1681/ASN.V76842

[24] Gellai M, Silverstein JH, Hwang JC, LaRochelle FT Jr, Valtin H (1984) Influence of vasopressin on renal hemodynamics in conscious Brattleboro rats. Am J Physiol 246: F819–827. 6742157 10.1152/ajprenal.1984.246.6.F819

[25] Bankir L, Bouby N, Trinh-Trang-Tan MM (1991) Vasopressin-dependent kidney hypertrophy: role of urinary concentration in protein-induced hypertrophy and in the progression of chronic renal failure. Am J Kidney Dis 17: 661–665. 2042645 10.1016/s0272-6386(12)80346-5

[26] Bouby N, Fernandes S (2003) Mild dehydration, vasopressin and the kidney: animal and human studies. Eur J Clin Nutr 57 Suppl 2S39–46. 14681712 10.1038/sj.ejcn.1601900

[27] Anastasio P, Cirillo M, Spitali L, Frangiosa A, Pollastro RM, et al (2001) Level of hydration and renal function in healthy humans. Kidney Int 60: 748–756. 11473658 10.1046/j.1523-1755.2001.060002748.x

[28] Perico N, Zoja C, Corna D, Rottoli D, Gaspari F, et al (2009) V1/V2 Vasopressin receptor antagonism potentiates the renoprotection of renin-angiotensin system inhibition in rats with renal mass reduction. Kidney Int 76: 960–967. 19625993 10.1038/ki.2009.267

[29] Bardoux P, Bruneval P, Heudes D, Bouby N, Bankir L (2003) Diabetes-induced albuminuria: role of antidiuretic hormone as revealed by chronic V2 receptor antagonism in rats. Nephrol Dial Transplant 18: 1755–1763. 12937221 10.1093/ndt/gfg277

